# Nutritional Profiles of *Ruditapes philippinarum* and the Complementary Food-Resource Potential of the Warm-Water Clam *Ruditapes variegatus*

**DOI:** 10.3390/foods15173038

**Published:** 2026-08-28

**Authors:** Tomoyasu Yamazaki, Kenji Okoshi

**Affiliations:** Toyo Institute of Food Technology, 23-2, 4-Chome, Minami-Hanayashiki, Kawanishi 666-0026, Hyogo, Japan; kenji_okoshi@shokuken.or.jp

**Keywords:** Manila clam, *Ruditapes variegatus*, food composition, amino acid profile, taurine, underutilized bivalve, complementary food resource, pooled sample

## Abstract

The sustained decline in Japanese Manila clam landings has increased interest in complementary bivalve food resources. This descriptive study compared one pooled soft-tissue analytical sample from each of four sources: *Ruditapes philippinarum* from Akkeshi, Mangoku-ura, and a commercial Chinese-origin source, and the warm-water clam *Ruditapes variegatus* from Ishigaki Island. Each pooled sample comprised approximately 250 g of soft tissue from at least 40 individuals; independent lot-level biological replication was not available, so no inferential statistical comparisons were made. Proximate composition, amino acids, taurine, vitamin-related compounds, minerals, and fatty acids were analyzed, and mitochondrial cytochrome c oxidase subunit I (COI) sequencing together with morphology confirmed the Ishigaki material as *R. variegatus*. Wet-weight total amino acid contents were 8.670, 6.800, 6.135, and 5.555 g/100 g in the Akkeshi, Ishigaki, Mangoku-ura, and Chinese-origin pooled samples, respectively. After normalization, total amino acid contents ranged from 507.0 to 559.4 mg/g dry matter and from 829.1 to 850.0 mg/g protein, indicating that much of the wet-weight contrast reflected differences in tissue moisture and protein concentration. Adult-reference amino acid scores ranged from 97.1 to 100 across the four composites. The Akkeshi pooled sample also showed comparatively high taurine and vitamin A-related values. Sea surface temperature and satellite chlorophyll-a data were used only to document source-region environmental context and were not treated as causal predictors of composition. Within the limits of this pooled-sample survey, the food-composition profile of taxonomically verified Ishigaki *R. variegatus* supports further replicated evaluation of this species as a complementary clam-like food resource.

## 1. Introduction

The Manila clam *Ruditapes philippinarum* is an important edible bivalve widely harvested from tidal flats and inner bays in Japan. National landings peaked at 160,424 t in 1983 and declined to 4439 t in 2024, only 2.8% of the peak value [1]. This long-term decline has increased interest in both the sustainable use of existing Manila clam resources and the evaluation of complementary bivalve food resources.

The decline in Manila clam landings has been attributed to multiple, interacting factors, including fishing pressure, collection of juveniles for seed, sediment disturbance, coastal reclamation, hypoxia, flooding, predation, disease, parasitism, and changes in food availability [2,3]. The present study does not attempt to identify the causes of this national resource decline; rather, the decline provides the fisheries and food-resource context for evaluating the composition of available clam materials.

Food composition in *R. philippinarum* is known to vary with season, physiological state, storage, and food environment. Previous studies have reported seasonal changes in organic acids [4], changes in organic acids during storage [5], variation in inosine monophosphate during frozen storage [6], seasonal changes in proximate composition [7], mineral composition [8], extract components relevant to flavor [9], and seasonal variation in amino acids, 5′-nucleotides, and lipids [10]. Feeding ecology also changes with body size after settlement, with diatom-specific DNA metabarcoding indicating greater use of benthic diatoms by smaller juveniles and greater use of planktonic diatoms by larger individuals [11]. These findings show that food-composition measurements should be interpreted in relation to sample history and biological context rather than assumed to represent fixed regional or species-level values.

Commercially distributed Manila clams present an additional provenance issue because country-of-origin information does not resolve harvest locality, culture status, holding history, or post-harvest handling. Stable-isotope and fatty-acid-based approaches have been used to improve geographical traceability of commercial *R. philippinarum* [12]. In addition, introduced *Ruditapes* lineages and hybridization with native *R. philippinarum* have been reported in Japan [13], emphasizing the importance of taxonomic verification when evaluating unfamiliar or introduced clam resources.

For the warm-water congener *Ruditapes variegata* (often treated as *R. variegatus* in taxonomic usage), Dong et al. [14] reported a broad range of nutrients in the edible portion and discussed its value as an aquaculture candidate. However, direct food-science comparisons that combine multi-component composition profiling, molecular confirmation of warm-water *Ruditapes* material, and normalization for moisture and protein remain limited. In particular, it remains unclear whether the measured composition of an underutilized warm-water *Ruditapes* sample falls within the range observed for commercially important Manila clam samples once simple concentration effects are considered.

Accordingly, we compared one pooled analytical sample of *R. philippinarum* from each of Akkeshi, Mangoku-ura, and a commercial Chinese-origin source with one pooled sample of *R. variegatus* from Ishigaki Island. Proximate composition, amino acids, taurine, vitamin-related compounds, minerals, and fatty acids were measured, while cytochrome c oxidase subunit I (COI) sequencing and morphology were used to verify the identity of the Ishigaki sample. Sea surface temperature and satellite-derived chlorophyll-a were retained only as concise descriptors of source-region context.

Because independent lot-level biological replication was not available, the study was designed as a descriptive pooled-sample survey rather than an inferential test of species or regional effects. It addressed three questions: (1) what food-composition profiles were observed in the four analyzed pooled samples; (2) to what extent did wet-weight differences in total amino acids narrow after dry-matter and protein normalization; and (3) did the taxonomically verified Ishigaki *R. variegatus* sample show a composition that justified further replicated evaluation as a complementary clam-like food resource?

## 2. Materials and Methods

### 2.1. Sample Collection and Identification

**Figure 1:**
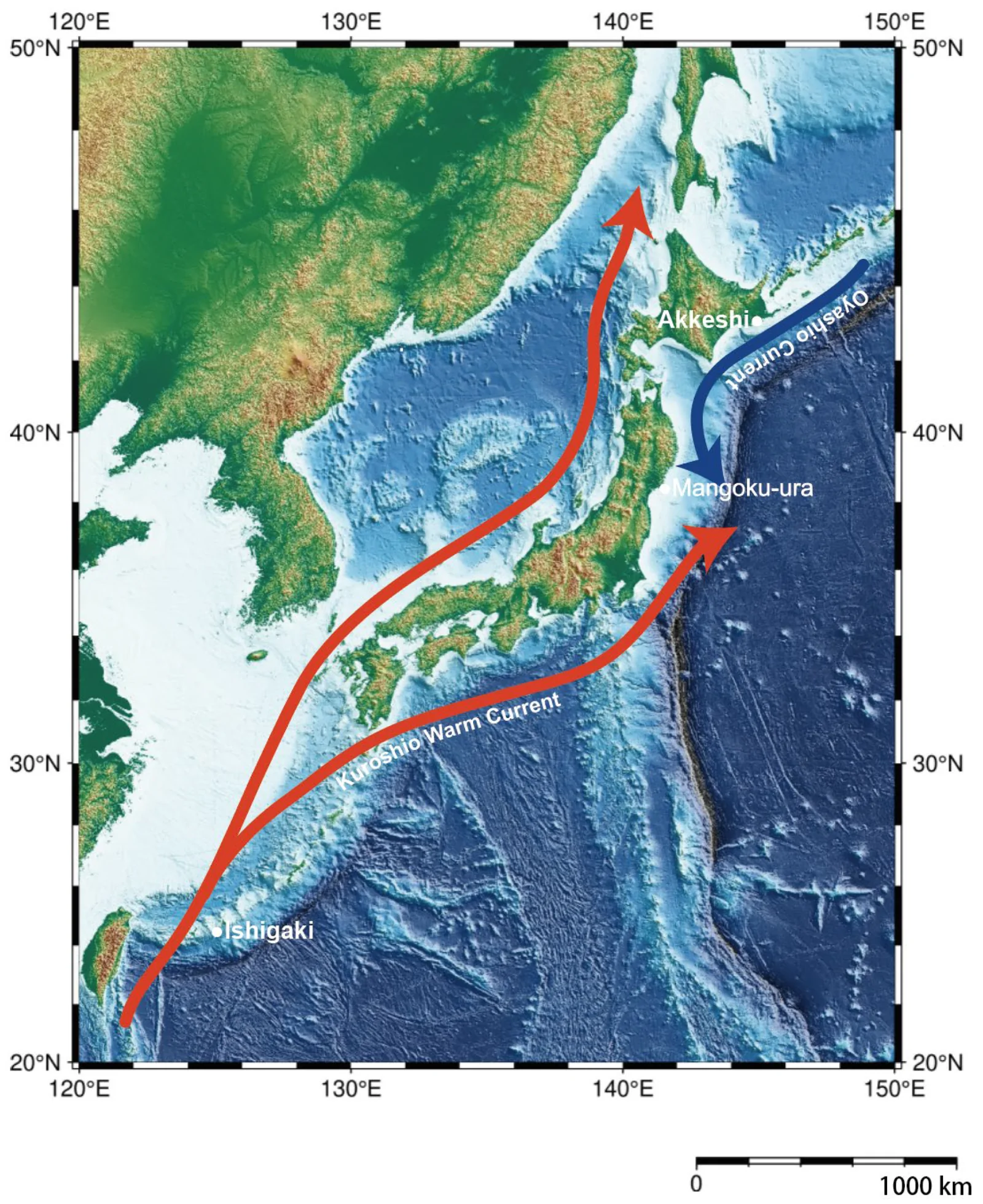
Source localities and oceanographic context. Map showing the source localities of Akkeshi, Mangoku-ura, and Ishigaki in relation to the major current systems around Japan. Red arrows indicate warm currents, and the blue arrow indicates the Oyashio Current. Background colors represent topographic and bathymetric relief and are shown for geographic context only. The Ishigaki sample represents *Ruditapes variegatus*, whereas the Akkeshi and Mangoku-ura samples represent *R. philippinarum*.

Bivalve materials were obtained from Akkeshi, Hokkaido; Mangoku-ura, Miyagi Prefecture; a commercial Chinese-origin source; and Ishigaki Island, Okinawa (Figure 1; Table 1). To comply with local fishery-right restrictions, the Akkeshi material was not collected directly from the fishery ground; instead, an Akkeshi-origin *Ruditapes philippinarum* sample was purchased at a local market in Akkeshi in January 2025. The Mangoku-ura and Ishigaki Island materials were collected for research in January 2025, and the Chinese-origin commercial sample was purchased from a supermarket in January 2025. Source coordinates were 43.05° N, 144.89° E for Akkeshi, 38.42° N, 141.38° E for Mangoku-ura, and 24.46° N, 124.22° E for Ishigaki Island. The exact harvest locality, wild or cultured status, holding history, and distribution history of the Chinese-origin commercial sample were unknown; it was therefore treated only as a commercial reference sample rather than as representative of a Chinese regional population.

For each source, approximately 250 g of pooled soft tissue was prepared as a single composite analytical sample. Based on an approximate soft-tissue mass of 6 g per clam, each composite included soft tissue from at least 40 individuals. Pooling provided sufficient material for the commissioned multi-component food analysis but did not constitute biological replication and does not estimate within-source variability. After collection or purchase in January 2025, soft tissues were removed and stored at −80 °C until the commissioned analysis; the samples were therefore analyzed after frozen storage rather than as fresh tissue. The same frozen-storage condition was applied to all four composites to minimize post-sampling enzymatic and microbial changes, although freezing and thawing may affect some low-molecular-weight constituents. Source-specific transportation conditions, exact time from collection or purchase to freezing, and the duration of frozen storage before analysis were not documented in the retained records available for this retrospective revision and are therefore not reconstructed here; this limitation is considered in Section 4.5.

For the Ishigaki material, molecular and morphological observations were used to confirm species identity. External morphology was examined with attention to shell appearance and siphon characters. The shell was broadly similar in appearance to that of the Manila clam, whereas siphon morphology was consistent with previously reported diagnostic features of *R. variegatus*: the inhalant and exhalant siphons of *R. philippinarum* are largely fused, whereas those of *R. variegatus* separate close to the base [15]. The siphon rim was also examined and was consistent with the reported morphology of *R. variegatus*. Representative siphonal morphology of the analyzed materials is shown in Supplementary Figure S4.

### 2.2. Nutritional Composition Analysis

Nutritional composition analyses were commissioned to the Japan Food Research Laboratories (JFRL). The analyzed endpoints comprised proximate composition (moisture, protein, lipid, ash, and carbohydrate), amino acid composition, taurine, vitamin A-related compounds, vitamin B12, minerals, and fatty acid composition. According to the JFRL analytical reports, moisture was determined by atmospheric-pressure heat drying, protein by the combustion method using a nitrogen-to-protein conversion factor of 6.25, lipid by Soxhlet extraction, and ash by direct ashing. Carbohydrate was calculated by difference as 100 − (moisture + protein + lipid + ash), and energy was calculated using factors of 4 kcal/g for protein, 9 kcal/g for lipid, and 4 kcal/g for carbohydrate. Sodium was measured by atomic absorption spectrophotometry, and salt equivalent was calculated as sodium × 2.54. Phosphorus, iron, calcium, magnesium, copper, zinc, and manganese were measured by inductively coupled plasma optical emission spectrometry. Vitamin A-related compounds (retinol, α-carotene, and β-carotene) were analyzed by high-performance liquid chromatography (HPLC), and vitamin A activity was expressed as retinol activity equivalents. Vitamin B12 was measured by a microbiological assay using *Lactobacillus delbrueckii* subsp. *lactis* (*L. leichmannii*) ATCC 7830. Amino acids were measured mainly using an amino acid autoanalyzer; tryptophan was determined by HPLC, and cystine was measured after performic acid oxidation followed by hydrochloric acid hydrolysis. Taurine was also measured using an amino acid autoanalyzer. The 18 quantified proteinogenic amino acids, excluding taurine, were summed as total amino acids; taurine was treated separately. Reported units were standardized to mg/100 g wet weight for analysis and visualization. Fatty acid composition was analyzed by gas chromatography as a separate endpoint of the commissioned food-composition analysis; because total fatty acids and most individual fatty acids were below the laboratory quantification limits in all four samples, these measurements were not used as a principal comparative endpoint. The Soxhlet procedure described above applies to the proximate-lipid determination. The retained analytical documentation identifies gas chromatography as the fatty-acid endpoint but does not provide sufficient detail on the preceding fatty-acid extraction/preparation to establish that it was identical to the proximate-lipid Soxhlet procedure; accordingly, the below-quantification fatty-acid results are not attributed to Soxhlet recovery. Because solvent-extraction recovery can depend on lipid class and extraction chemistry, the proximate lipid values are interpreted operationally as values obtained by the commissioned Soxhlet method rather than as exhaustive recovery of all lipid classes. The commissioned reports specified analytical techniques but did not consistently provide instrument model information; only documented analytical principles are therefore reported here.

Each source was represented by one pooled analytical composite; independent lot-level biological replication was not available. Therefore, no inferential statistical tests, population means, or species- or region-level significance claims were calculated. The results are reported as descriptive contrasts among the four analyzed composites. To examine the contribution of tissue concentration to wet-weight total amino acid values, total amino acids were additionally normalized to dry matter and protein. Dry-matter-normalized values were calculated as total amino acids divided by dry matter (100 − moisture content) and are expressed as mg/g dry matter. Protein-normalized values were calculated as total amino acids divided by protein content and are expressed as mg/g protein.

To add a compositional protein-quality perspective, indispensable amino acid reference ratios were calculated using the FAO adult (>18 years) amino acid scoring pattern (mg/g protein: histidine 15, isoleucine 30, leucine 59, lysine 45, methionine + cystine 22, phenylalanine + tyrosine 38, threonine 23, tryptophan 6.0, and valine 39) [16]. Amino acid concentrations were first expressed as mg/g measured protein. For each indispensable amino acid or amino acid pair, the reference ratio was calculated as the observed value divided by the FAO reference value × 100. The lowest reference ratio was taken as the uncorrected amino acid score; values above 100 were capped at 100 for summary reporting. Because protein digestibility was not measured, these values are compositional amino acid scores and should not be interpreted as PDCAAS or DIAAS.

An exploratory principal component analysis (PCA) was performed on the 18 quantified amino acids after normalization to measured protein (mg/g protein) and z-score standardization across the four pooled composites. PCA was conducted in Python version 3.13.5 using scikit-learn version 1.8.0. The analysis was used only to visualize sample-level profile structure; with only four pooled composites, it was not treated as inferential evidence of group separation, and no supervised PLS-DA was performed.

### 2.3. Oceanographic Data Sources and Processing

Regional environmental context was summarized using sea surface temperature (SST) data from the Japan Meteorological Agency [17] and GCOM-C/SGLI Level-3 monthly chlorophyll-a products from the Japan Aerospace Exploration Agency [18]. SST was represented by Kushiro Area 122 for Akkeshi, Southern Iwate Area 133 for Mangoku-ura, and Southeast of Ishigaki Island Area 709 for Ishigaki; monthly means were calculated from daily data for 1982–2025. Seasonal chlorophyll-a means for 2024 were calculated from monthly products, and site values were estimated as 3 × 3 pixel local means to reduce coastal masking effects. Detailed SST contours and seasonal chlorophyll-a maps are provided as Supplementary Figures S1 and S2, respectively. Seasonal site-level chlorophyll-a values are listed in Supplementary Table S2, and the specific GCOM-C/SGLI source files are listed in Supplementary Table S3. These variables were used only as contextual descriptors and were not statistically related to food-composition values.

### 2.4. COI Sequencing, BLASTN Search, Phylogenetic Analysis, and Genetic Distance Analysis

Molecular identification of the Ishigaki *R. variegatus* material was performed using the mitochondrial cytochrome c oxidase subunit I (COI) region. Fifteen specimens were sequenced. For each specimen, an approximately 3 × 3 × 3 mm piece of adductor muscle tissue was used for DNA extraction. The exact tissue mass was not measured or recorded. Tissue was freeze-dried using a VD-250R Freeze Dryer (TAITEC, Koshigaya, Japan) and homogenized with a Multi-Beads Shocker (Yasui Kikai, Osaka, Japan) at 1500 rpm for 2 min. DNA was extracted after lysis with Lysis Solution F (Nippon Gene, Tokyo, Japan) and Proteinase K (TaKaRa, Kusatsu, Japan) at 56 °C for 10 min, followed by centrifugation at 12,000× *g* for 2 min and purification using a Lab-Aid 824s DNA Extraction Kit (ZEESAN, Xiamen, China). DNA concentration was measured using a Synergy LX microplate reader (Agilent Technologies, Santa Clara, CA, USA) and QuantiFluor dsDNA System (Promega, Madison, WI, USA).

The COI fragment was amplified using the primers COIF-ALT (5′-ACAAATCAYAARGAYATYGG-3′) and COIR-ALT (5′-TTCAGGRTGNCCRAARAAYCA-3′). PCR was conducted using KOD FX Neo (TOYOBO, Osaka, Japan) in a 20 μL reaction mixture containing 2× PCR Buffer for KOD FX Neo, dNTPs, primers, template DNA, polymerase, and nuclease-free water. The PCR profile consisted of an initial denaturation at 94 °C for 2 min, followed by 38 cycles of 98 °C for 10 s, 50 °C for 30 s, and 68 °C for 45 s, with a final extension at 68 °C for 7 min. PCR products were purified using VAHTS DNA Clean Beads (Vazyme, Nanjing, China). Product concentration was measured using a Synergy H1 microplate reader (Agilent Technologies) and QuantiFluor dsDNA System, and product quality was checked using a Fragment Analyzer and dsDNA 915 Reagent Kit (Agilent Technologies). Sanger sequencing was outsourced to Macrogen Japan.

Forward and reverse reads were visually checked, low-quality regions were trimmed, and contigs were assembled and aligned in MEGA version 10.2.6 [19]. The assembled COI sequences were compared with the NCBI nucleotide database using BLASTN version 2.15.0 [20]. Although COI sequences were obtained from 15 Ishigaki specimens, 13 specimens were used for the final phylogenetic analysis; two specimens were excluded during alignment to retain a longer homologous region across the dataset. The final alignment used for phylogenetic tree construction was 653 bp. The phylogenetic tree was inferred in MEGA version 10.2.6 using the maximum-likelihood method with the Kimura 2-parameter model, gamma-distributed rate variation with invariant sites (G + I), five discrete gamma categories, complete deletion of sites containing gaps or missing data, nearest-neighbor-interchange (NNI) as the ML heuristic method, and an automatically generated neighbor-joining initial tree. Branch support was evaluated with the bootstrap method using 10,000 replicates [21]. Pairwise genetic distances among COI sequences were calculated in MEGA version 10.2.6 using the Kimura 2-parameter model. Codon positions included were the 1st, 2nd, 3rd, and noncoding positions. All positions containing gaps and missing data were eliminated, resulting in a final dataset of 651 positions for genetic distance analysis.

## 3. Results

### 3.1. Descriptive Food-Composition Profiles

**Figure 2:**
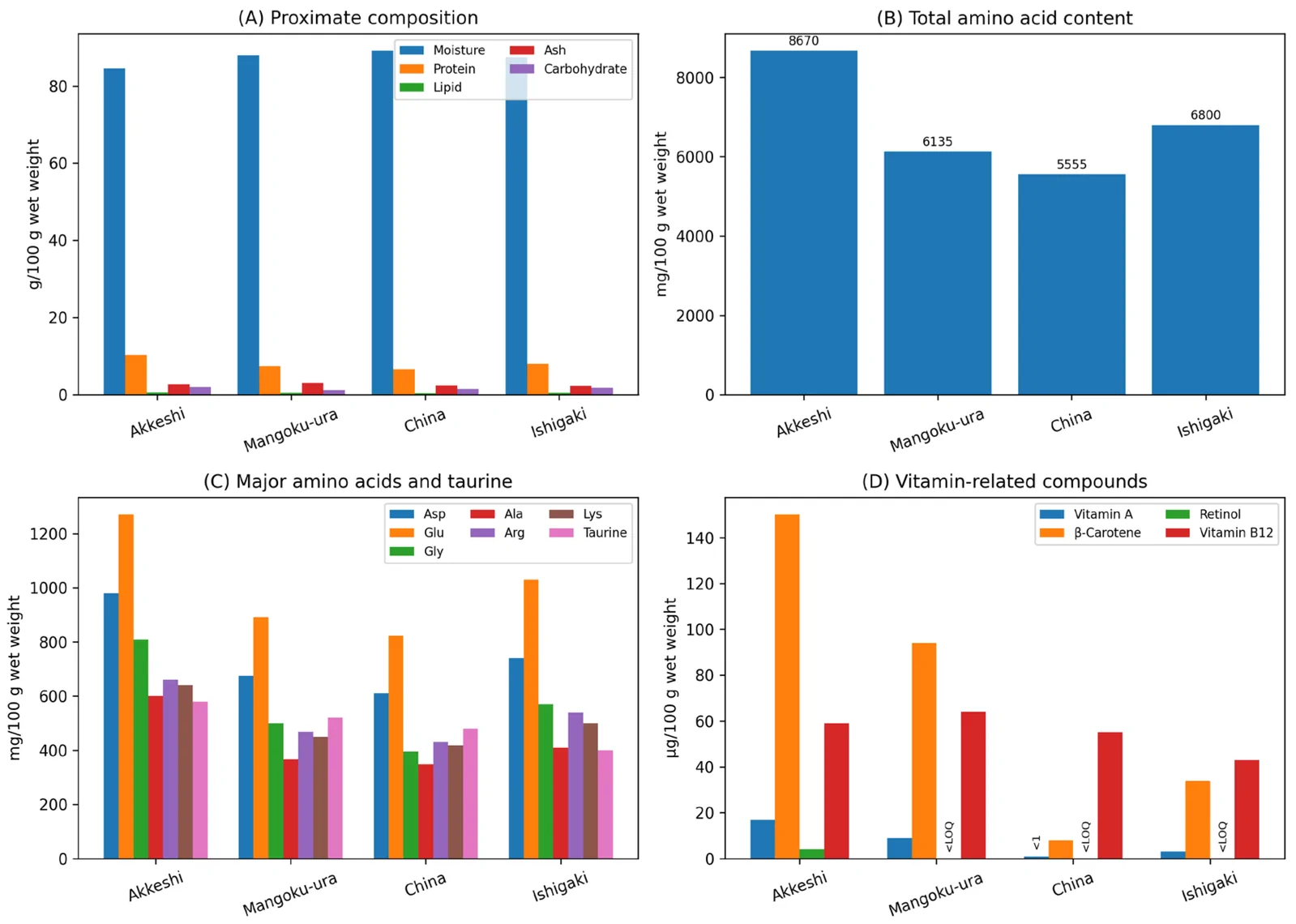
Food-composition profiles of the four analyzed pooled clam samples. (**A**) Proximate composition (g/100 g wet weight). (**B**) Total amino acid content (mg/100 g wet weight), calculated as the sum of the 18 quantified proteinogenic amino acids; taurine was analyzed separately and is not included in this total. (**C**) Major amino acids and taurine (mg/100 g wet weight). (**D**) Vitamin-related compounds (μg/100 g wet weight), including vitamin A, β-carotene, retinol, and vitamin B12. The Ishigaki pooled sample represents *Ruditapes variegatus*; the other three samples represent *R. philippinarum*. Values are descriptive measurements of single pooled analytical composites. In panel (**D**), retinol values for Mangoku-ura, the Chinese-origin commercial composite, and Ishigaki were below the analytical detection/quantification limit and should be interpreted as <LOQ rather than zero.

The measured food-composition profiles differed numerically among the four pooled analytical samples (Figure 2A–D). The Akkeshi composite had the lowest moisture content and the highest measured protein content. Wet-weight total amino acid contents were 8.670 g/100 g in Akkeshi, 6.800 g/100 g in Ishigaki *R. variegatus*, 6.135 g/100 g in Mangoku-ura, and 5.555 g/100 g in the Chinese-origin commercial composite (Figure 2B). These values are descriptive measurements of the analyzed composites; no statistical significance or population-level differences are implied.

The Akkeshi composite also had the highest measured values for several abundant amino acids, including aspartic acid, glutamic acid, glycine, alanine, arginine, and lysine, and showed a comparatively high taurine value (Figure 2C). The analysis quantified total amino acids and did not distinguish the free fraction from protein-bound amino acids. For vitamin-related compounds, the Akkeshi composite showed the highest measured β-carotene and vitamin A-related values; retinol was quantifiable in the Akkeshi sample, whereas the other samples were reported below the analytical quantification limit (Figure 2D). Mineral values are provided in Supplementary Table S1.

Normalization substantially narrowed the range of total amino acid values. Dry-matter-normalized values ranged from 507.0 mg/g in Mangoku-ura to 559.4 mg/g in Akkeshi; Ishigaki and the Chinese-origin composite showed 539.7 and 509.6 mg/g, respectively. Protein-normalized values ranged from 829.1 mg/g protein in Mangoku-ura to 850.0 mg/g protein in Ishigaki; Akkeshi and the Chinese-origin composite both showed 841.7 mg/g protein. Thus, the wider contrast on an edible wet-weight basis was strongly influenced by differences in tissue moisture and protein concentration. Protein normalization showed a narrow range of total amino acid content per gram of measured protein across these four composites, but it does not by itself establish similarity in the intrinsic amino acid composition of their proteins.

Adult-reference indispensable amino acid balance was high in all four composites (Supplementary Table S4). Valine yielded the lowest reference ratio in each sample. The uncorrected amino acid scores were 97.1 for Akkeshi, 99.8 for Mangoku-ura, 100 for the Chinese-origin commercial composite (capped at 100; raw minimum ratio 101.4), and 99.4 for Ishigaki. These values indicate that the measured indispensable amino acid profiles were close to or met the FAO adult reference pattern within the analyzed composites, without correction for digestibility.

Exploratory PCA of the 18 protein-normalized amino acid variables showed that PC1 and PC2 explained 59.5% and 28.9% of the variance, respectively (88.5% cumulatively; Supplementary Figure S3). Akkeshi was displaced primarily along PC1, whereas Mangoku-ura and Ishigaki were separated mainly along PC2; the Chinese-origin commercial composite occupied a distinct positive-PC1 position closer to the PC2 origin. Because the PCA contains only four pooled composites, these positions describe the measured samples and are not replicated source clusters.

### 3.2. Oceanographic Context

The source regions spanned a marked thermal gradient (Supplementary Figure S1): Akkeshi was the coldest, Mangoku-ura showed an intermediate seasonal regime, and Ishigaki remained warm throughout the year. Surface chlorophyll-a was higher around Akkeshi than around Mangoku-ura and Ishigaki (Supplementary Figure S2; Supplementary Table S2). Seasonal site-level values were 2.423–4.100 mg m^−3^ at Akkeshi, 1.162–2.215 mg m^−3^ at Mangoku-ura, and 0.646–1.272 mg m^−3^ at Ishigaki. These environmental measurements describe source-region context only and were not tested as explanatory variables for food composition.

### 3.3. Molecular Identification of the Ishigaki R. variegatus Sample

COI sequences were successfully obtained from 15 Ishigaki specimens. After trimming and assembly, the final alignment length for phylogenetic analysis was 653 bp. To maximize the number of homologous nucleotide positions used in the phylogenetic tree, 13 of the 15 specimens were retained for tree construction, whereas two specimens with shorter comparable sequence regions were excluded from the final alignment.

**Figure 3:**
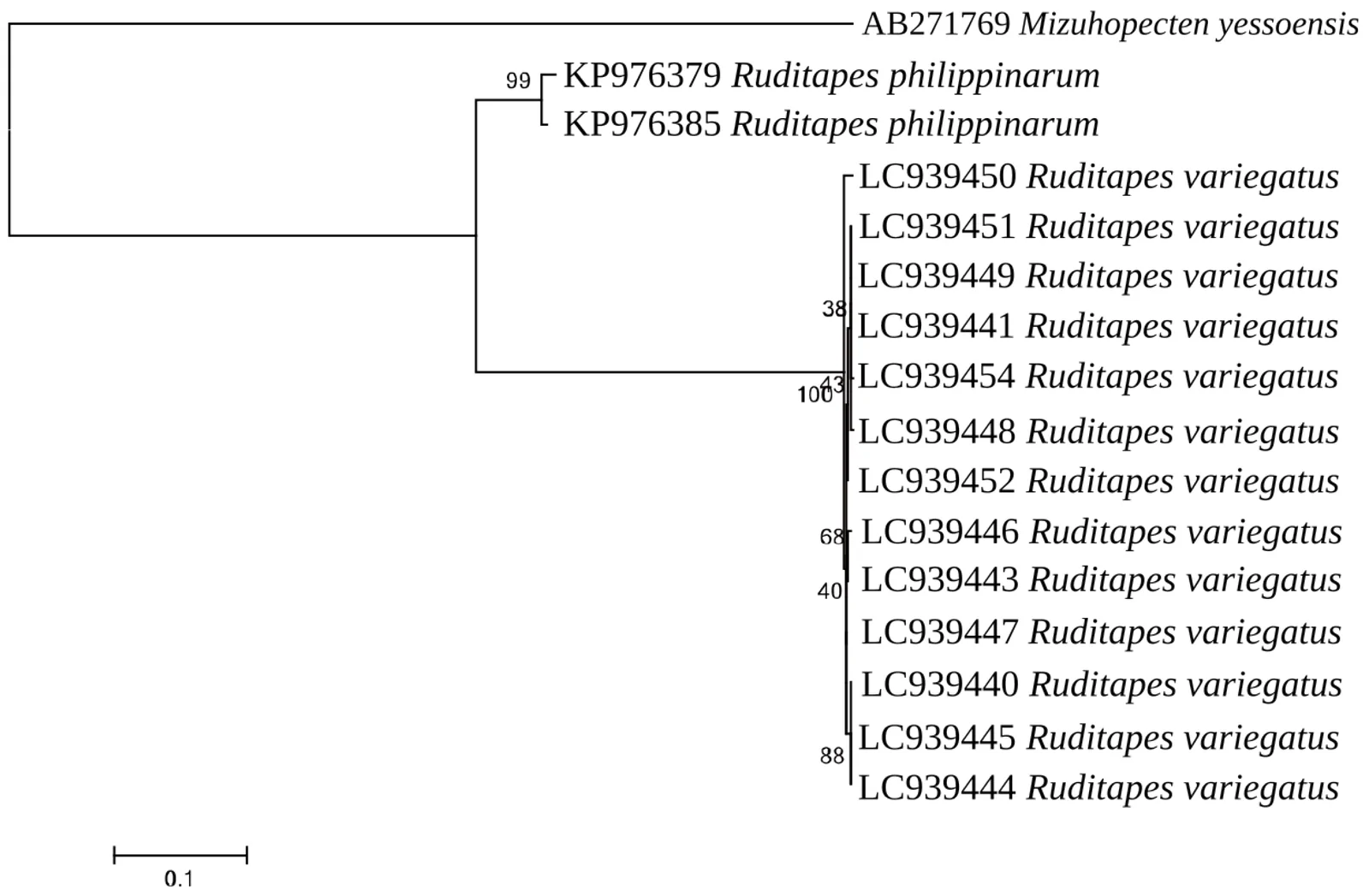
COI phylogenetic tree of the Ishigaki *R. variegatus* samples and reference sequences. The tree was constructed from a 653-bp alignment using 13 Ishigaki specimens and reference sequences of *Ruditapes philippinarum* and related taxa. Two of the 15 sequenced Ishigaki specimens were excluded during alignment to retain a longer homologous region. The tree was inferred using the maximum-likelihood method, and bootstrap support values are shown at the nodes. *Mizuhopecten yessoensis* was used as the outgroup. The Ishigaki sequences formed a clade separated from the *R. philippinarum* references and clustered with sequences corresponding to the *R. variegatus*/*R. variegata* complex. DDBJ accession numbers are LC939440–LC939454.

BLASTN searches showed high similarity to reference sequences annotated as *Ruditapes variegatus*, *Ruditapes variegata*, *Venerupis variegata*, or *Venerupis aspera*, with top-hit identities generally exceeding 99% for most specimens. In the maximum-likelihood COI tree, the 13 Ishigaki sequences formed a cluster separated from the *R. philippinarum* references and grouped with reference sequences corresponding to the *R. variegatus*/*R. variegata* complex (Figure 3). Together with the siphon morphology described above, these molecular results support treatment of the Ishigaki material as *R. variegatus* in this study. The newly generated COI sequences were deposited in DDBJ under accession numbers LC939440–LC939454.

The mean K2P genetic distance between the Ishigaki *R. variegatus* sequences and the two *R. philippinarum* reference sequences was 0.2975 (range 0.293–0.300), whereas the mean distance among Ishigaki *R. variegatus* sequences was 0.0053 (range 0.000–0.012). These measurements confirm clear COI-level differentiation between the Ishigaki material and the *R. philippinarum* references.

## 4. Discussion

### 4.1. Interpretation of the Measured Food-Composition Profiles

On an edible wet-weight basis, the Akkeshi composite had the highest measured total amino acid content (8.670 g/100 g), followed by Ishigaki *R. variegatus* (6.800 g/100 g), Mangoku-ura (6.135 g/100 g), and the Chinese-origin commercial composite (5.555 g/100 g). These values provide a direct description of the analyzed materials but should not be interpreted as estimates of regional or species means because each source was represented by only one pooled analytical composite.

Normalization materially changed the interpretation. The range narrowed to 507.0–559.4 mg/g dry matter and 829.1–850.0 mg/g protein. Thus, much of the difference observed per 100 g of edible tissue reflected variation in moisture and protein concentration rather than a large difference in total amino acid content per gram of measured protein. Both expressions are useful from a food-science perspective: wet-weight values describe nutrient density in the tissue as consumed, whereas dry-matter and protein normalization provide concentration-adjusted descriptions. Protein normalization alone does not establish that the intrinsic amino acid composition of the proteins is equivalent among samples.

The adult-reference amino acid scores provide information that total protein normalization alone cannot: they assess the balance of indispensable amino acids relative to a defined human requirement pattern. Scores of 97.1–100 across the four composites, with valine as the lowest-ratio amino acid in each case, indicate broadly similar indispensable-amino-acid adequacy at the sample level. The exploratory PCA nevertheless showed that the complete protein-normalized amino acid profiles were not identical. These analyses therefore complement, rather than replace, the wet-weight and concentration-normalized comparisons. Because digestibility and independent biological replication were unavailable, the scores and PCA should not be interpreted as estimates of protein quality or species-level differences.

The Akkeshi composite also showed comparatively high measured taurine and vitamin A-related values. Several amino acids associated with shellfish flavor chemistry, including glutamic acid, aspartic acid, glycine, and alanine, were abundant; however, the commissioned analysis quantified total amino acids and did not separate the free amino acid fraction that contributes directly to taste. The present data therefore describe composition but cannot establish sensory superiority or differences in flavor intensity.

### 4.2. Oceanographic Context: Scope and Interpretation

The environmental analysis confirmed that the source regions occupied markedly different long-term thermal settings (Supplementary Figure S1). Temperature is known to influence physiological energetics in *R. philippinarum* [22,23,24,25], but the present study did not collect replicated nutritional samples across temperatures and therefore does not test temperature effects on food composition.

Seasonal surface chlorophyll-a was also higher around Akkeshi than around Ishigaki (Supplementary Figure S2; Supplementary Table S2), consistent with broad differences between Oyashio- and Kuroshio-influenced waters [26,27,28,29,30,31,32,33,34,35]. However, surface chlorophyll-a does not measure the material actually ingested by benthic clams, and phytoplankton composition, benthic microalgae, diet, and pigment profiles were not measured simultaneously with the food-composition samples. Consequently, the environmental datasets are retained only to document source-region context and to motivate hypotheses for replicated future work; they are not evidence that SST or chlorophyll-a caused the observed compositional ranking.

### 4.3. Food-Resource Relevance of the Ishigaki R. variegatus Sample

The Ishigaki material was molecularly and morphologically verified as *R. variegatus* rather than treated as a southern population of Manila clam. The mean COI K2P distance between the Ishigaki sequences and *R. philippinarum* references was 0.2975, compared with 0.0053 among Ishigaki sequences. This verification is important for food-resource evaluation because *R. variegatus* resembles Manila clam externally but is a distinct taxon requiring accurate identification and labeling.

The Ishigaki pooled sample contained 6.800 g/100 g total amino acids on a wet-weight basis and 850.0 mg/g protein after protein normalization. These values fell within the range measured for the three *R. philippinarum* pooled samples. This sample-level observation provides a basis for further replicated evaluation, but it is not evidence of species-level nutritional equivalence or superiority.

External resemblance to Manila clam may facilitate recognition as a clam-like food, but consumer acceptance cannot be inferred from appearance. Development as a food resource would require replicated evaluation of edible yield, composition across seasons and lots, safety, sensory properties, supply feasibility, labeling, and ecological effects of harvest or cultivation.

Dong et al. [14] also reported a broad range of nutrients in *Ruditapes variegata* and discussed its potential as an aquaculture species. Together with the present taxonomic verification and descriptive food-composition data, that report supports targeted follow-up studies of warm-water *Ruditapes* as a complementary resource. The present study does not establish *R. variegatus* as a direct substitute for *R. philippinarum*.

### 4.4. Interpretation of the Commercial Chinese-Origin Reference Sample

The commercial Chinese-origin composite had the lowest measured wet-weight total amino acid value among the four analyzed samples, but this observation cannot be generalized to Chinese *R. philippinarum*. The exact harvest locality, wild or cultured status, holding history, and distribution conditions were unavailable. Accordingly, this sample is retained only as a commercial reference category that illustrates the interpretive limitations of market-origin labeling, rather than as a regional comparator.

For commercially distributed clams, country-of-origin information alone does not resolve habitat or post-harvest history. Stable-isotope, fatty-acid, and compound-specific isotope approaches can improve geographical traceability [12]. Future studies that combine such provenance tools with replicated food-composition sampling would help separate biological origin, environment, and post-harvest handling.

### 4.5. Scope of Inference and Priorities for Validation

The principal limitation is the absence of independent lot-level biological replication. Pooling material from at least approximately 40 individuals yielded one analytical composite per source but did not estimate within-source variability. Consequently, the reported values and normalized contrasts describe the four analyzed composites only and do not support inferential statistics, regional means, or species-level claims. Additional limitations are the lack of simultaneous environmental measurements, incomplete provenance of the commercial Chinese-origin sample, absence of retained source-specific transport/time-to-freezing and frozen-storage-duration records, fatty acid values below quantification limits with incomplete documentation of the fatty-acid preparation step, and the use of total rather than free amino acid measurements.

The most informative validation design would include multiple independent lots and seasons per locality, standardized post-harvest handling, and concurrent measurements of environmental and food variables. Expanded analyses of free amino acids, organic acids, nucleotide-related compounds, glycogen, pigments, fatty acids, edible yield, safety, and sensory properties would permit a more complete nutritional and food-quality characterization. Within its descriptive scope, the present study provides baseline food-composition data, demonstrates the importance of moisture/protein normalization, and establishes the identity of the Ishigaki warm-water *Ruditapes* material for future replicated evaluation.

Within these limits, integrating food-composition measurements with moisture and protein normalization, molecular species confirmation, and clearly delimited environmental context provides a more informative description than a component inventory alone. The analysis distinguishes sample-level wet-weight contrasts that are strongly influenced by concentration effects and provides a taxonomically verified *R. variegatus* composite for subsequent replicated food-resource evaluation.

## 5. Conclusions

This descriptive study compared single pooled analytical composites from four clam sources. The Akkeshi *R. philippinarum* composite had the highest measured wet-weight total amino acid content (8.670 g/100 g) and comparatively high taurine and vitamin A-related values, but the narrower dry-matter- and protein-normalized ranges showed that much of the wet-weight contrast reflected tissue moisture and protein concentration. The taxonomically verified Ishigaki *R. variegatus* composite contained 6.800 g/100 g total amino acids and 850.0 mg/g protein after normalization, values within the range of the analyzed *R. philippinarum* composites. These sample-level observations support further replicated evaluation of *R. variegatus* as a complementary clam-like food resource but do not establish species- or region-level nutritional differences. Independent lot and seasonal replication, standardized handling, broader taste- and quality-related chemistry, safety assessment, sensory testing, and production studies are required to determine its practical food value. Adult-reference amino acid scores were 97.1–100 across the four composites, and exploratory PCA of protein-normalized amino acid profiles visualized additional sample-level compositional structure; both analyses remain descriptive because digestibility and independent biological replication were not available.

## Figures and Tables

**Table 1:** Sample information and oceanographic classification.

Sample	Source/Locality	Latitude	Longitude	Taxon	Oceanographic Classification/Notes
Akkeshi	Akkeshi, Hokkaido, Japan	43.05° N	144.89° E	*Ruditapes philippinarum*	Akkeshi-origin sample purchased in January 2025 at a local market in Akkeshi, Hokkaido; direct collection from the fishery ground was not conducted because of local fishery-right restrictions; Coastal Oyashio-influenced region; represented by JMA Kushiro Area 122 for SST analysis.
Mangoku-ura	Mangoku-ura, Miyagi Prefecture, Japan	38.42° N	141.38° E	*Ruditapes philippinarum*	Collected for research in January 2025; mixed Oyashio–Kuroshio coastal region; represented by JMA Southern Iwate Area 133 for SST analysis.
China	Commercial Chinese-origin sample	—	—	*Ruditapes philippinarum*	Purchased in January 2025 as a commercial imported reference sample; exact harvest area, wild/cultured status, holding history, and distribution history unknown.
Ishigaki	Ishigaki Island, Okinawa, Japan	24.46° N	124.22° E	*Ruditapes variegatus*	Collected for research in January 2025; subtropical Kuroshio-influenced region; represented by JMA Southeast of Ishigaki Island Area 709 for SST analysis; identity supported by COI and morphology.

## Data Availability

The original contributions presented in this study are included in the article/Supplementary Materials. Further inquiries can be directed to the corresponding author.

## Supplementary Materials

The following supporting information can be downloaded at: https://www.mdpi.com/article/10.3390/foods15173038/s1, Figure S1. Monthly sea surface temperature contours for the regions corresponding to the source areas. Monthly mean sea surface temperatures were calculated from daily Japan Meteorological Agency data from 1982 to 2025 [17]. (A) Akkeshi, represented by Kushiro Area 122; (B) Mangoku-ura, represented by Southern Iwate Area 133; and (C) Ishigaki, represented by Southeast of Ishigaki Island Area 709. The shared color scale and isotherms show seasonal and long-term contrasts among regions. Figure S2. Seasonal chlorophyll-a distribution around Japan derived from GCOM-C/SGLI satellite observations in 2024 [18]. Seasonal means were calculated for spring (March–May), summer (June–August), autumn (September–November), and winter (January, February, and December). Source localities are indicated by black circles, and chlorophyll-a concentrations are shown on a logarithmic scale (mg m^−3^). Figure S3. Exploratory principal component analysis (PCA) of protein-normalized amino acid profiles in the four pooled analytical composites. The 18 quantified amino acids were expressed as mg/g measured protein and z-score standardized before PCA. PC1 and PC2 explained 59.5% and 28.9% of the variance, respectively (88.5% cumulatively). The score plot is descriptive only; each point represents a single pooled source composite and does not constitute a replicated group. Figure S4. Representative siphonal morphology of *Ruditapes philippinarum* and *Ruditapes variegatus*. (A) *Ruditapes philippinarum* from the same commercial Chinese-origin lot used for the food-composition analysis. (B) *Ruditapes variegatus* from the same Ishigaki Island lot used for the food-composition analysis and COI sequencing. The two taxa were compared with reference to the diagnostic siphonal characters described by Sagara [15]. In *R. philippinarum*, the inhalant and exhalant siphons remain fused over most of their length and separate mainly toward the distal region, whereas in *R. variegatus* the two siphons separate closer to the base. Differences in the morphology of the marginal siphonal tentacles are also visible. Scale bars = 1 cm. Molecular identification of the Ishigaki *R. variegatus* material was independently supported by COI sequence analysis. Table S1. Mineral composition of the four analyzed pooled clam samples. Table S2. Seasonal site-level chlorophyll-a values extracted from GCOM-C/SGLI data. Table S3. GCOM-C/SGLI Level-3 monthly chlorophyll-a (CHLAF) files used for seasonal mapping. Table S4. Adult-reference indispensable amino acid composition and amino acid scores of the four analyzed pooled clam samples. Table S5. Raw and protein-normalized amino acid dataset used for amino acid score calculation and exploratory PCA.
