# Supplementary material for: Nutritional Profiles of Ruditapes philippinarum and the Complementary Food-Resource Potential of the Warm-Water Clam Ruditapes variegatus
Source: Foods. 2026 Aug 28;15(17):3038. doi: 10.3390/foods15173038 (PMC13565222; doi:10.3390/foods15173038)
Supplement: Supplementary file 1 [file foods-15-03038-s001.zip › Figure_S1-S4.pdf]

Supplementary Figures

Nutritional Profiles of *Ruditapes philippinarum* and the Complementary Food-Resource Potential of the Warm-Water Clam Ruditapes variegatus

Tomoyasu Yamazaki and Kenji Okoshi

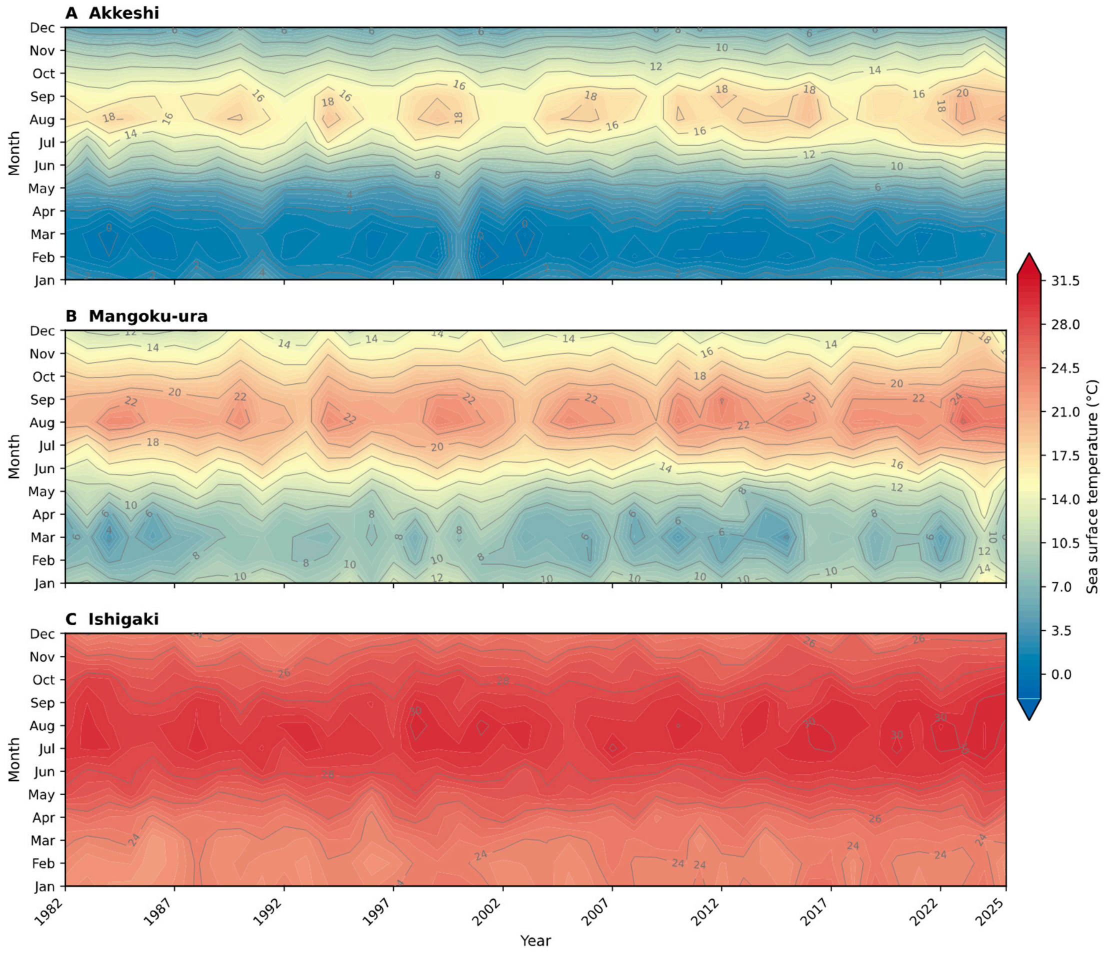

Figure S1. Monthly sea surface temperature contours for the regions corresponding to the source areas. Monthly mean sea surface temperatures were calculated from daily Japan Meteorological Agency data from 1982 to 2025 [17]. (A) Akkeshi, represented by Kushiro Area 122; (B) Mangoku-ura, represented by Southern Iwate Area 133; and (C) Ishigaki, represented by Southeast of Ishigaki Island Area 709. The shared color scale and isotherms show seasonal and long-term contrasts among regions.

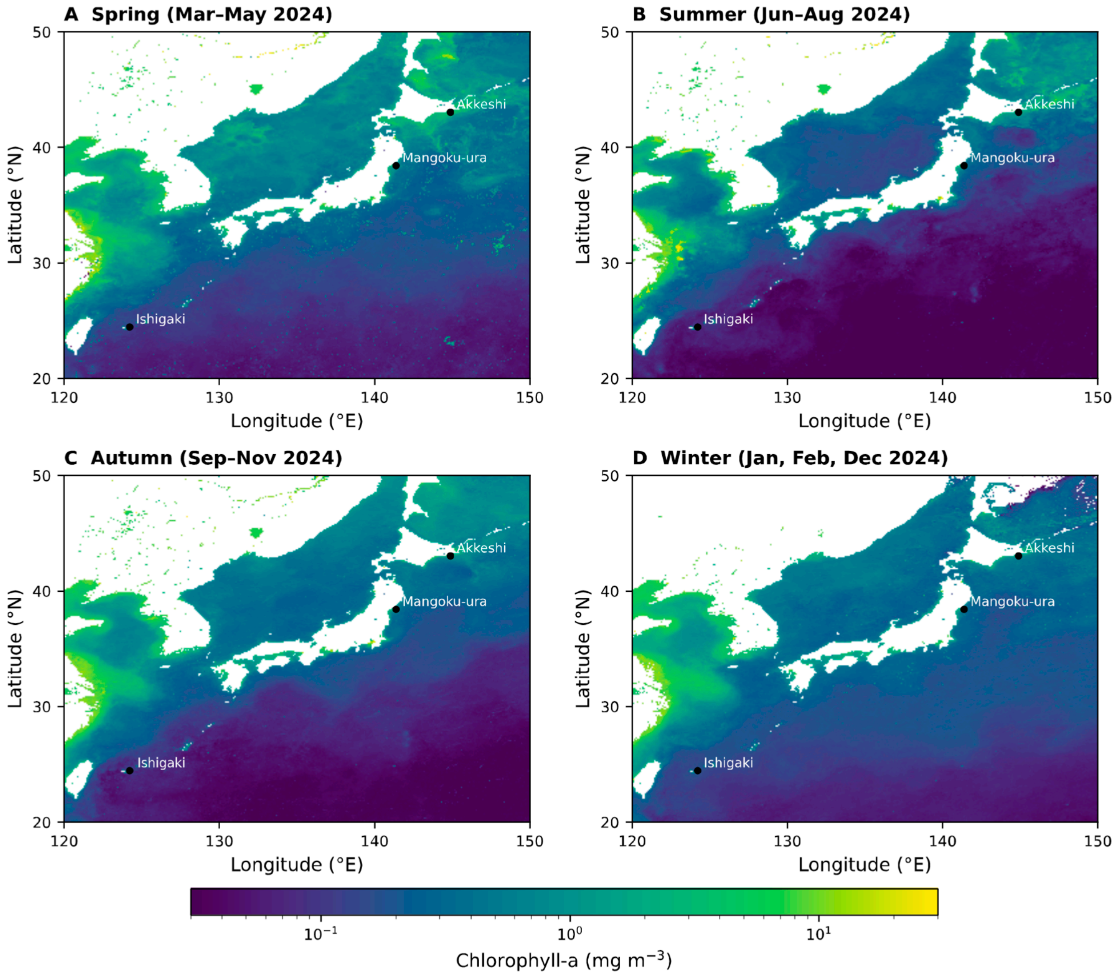

Figure S2. Seasonal chlorophyll-a distribution around Japan derived from GCOM-C/SGLI satellite observations in 2024 [18]. Seasonal means were calculated for spring (March–May), summer (June–August), autumn (September–November), and winter (January, February, and December). Source localities are indicated by black circles, and chlorophyll-a concentrations are shown on a logarithmic scale (mg m^{-3}).

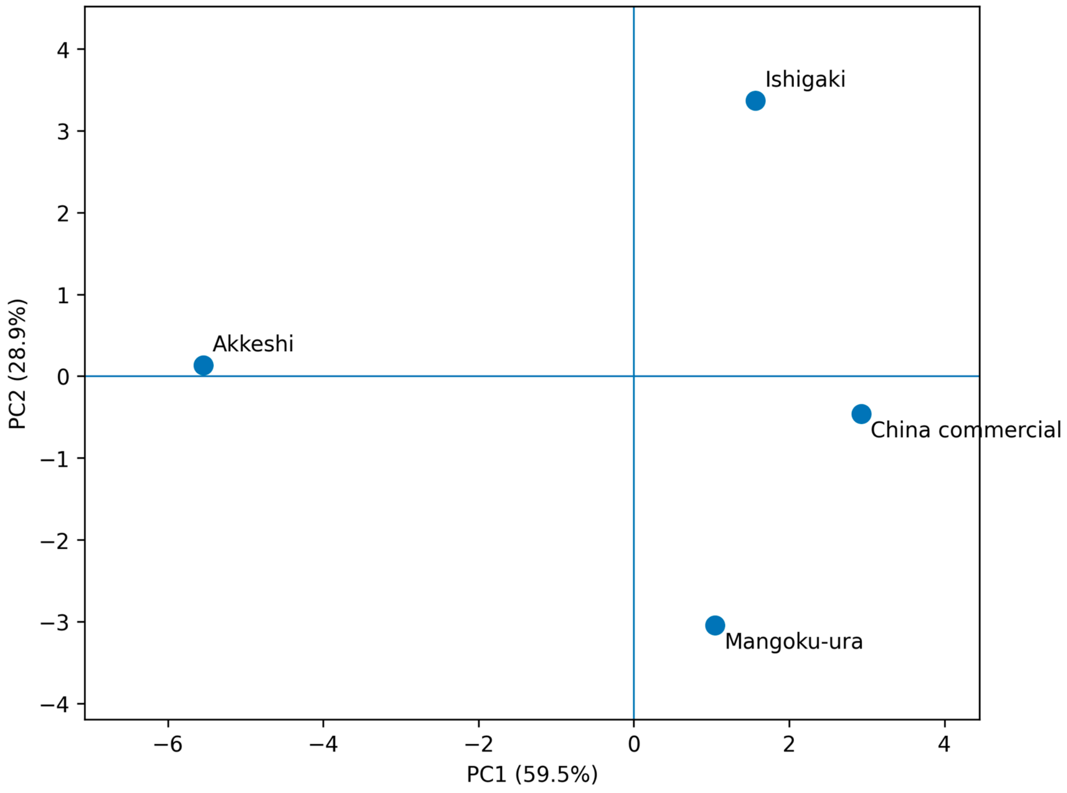

Figure S3. Exploratory principal component analysis (PCA) of protein-normalized amino acid profiles in the four pooled analytical composites. The 18 quantified amino acids were expressed as mg/g measured protein and z-score standardized before PCA. PC1 and PC2 explained 59.5% and 28.9% of the variance, respectively (88.5% cumulatively). The score plot is descriptive only; each point represents a single pooled source composite and does not constitute a replicated group.

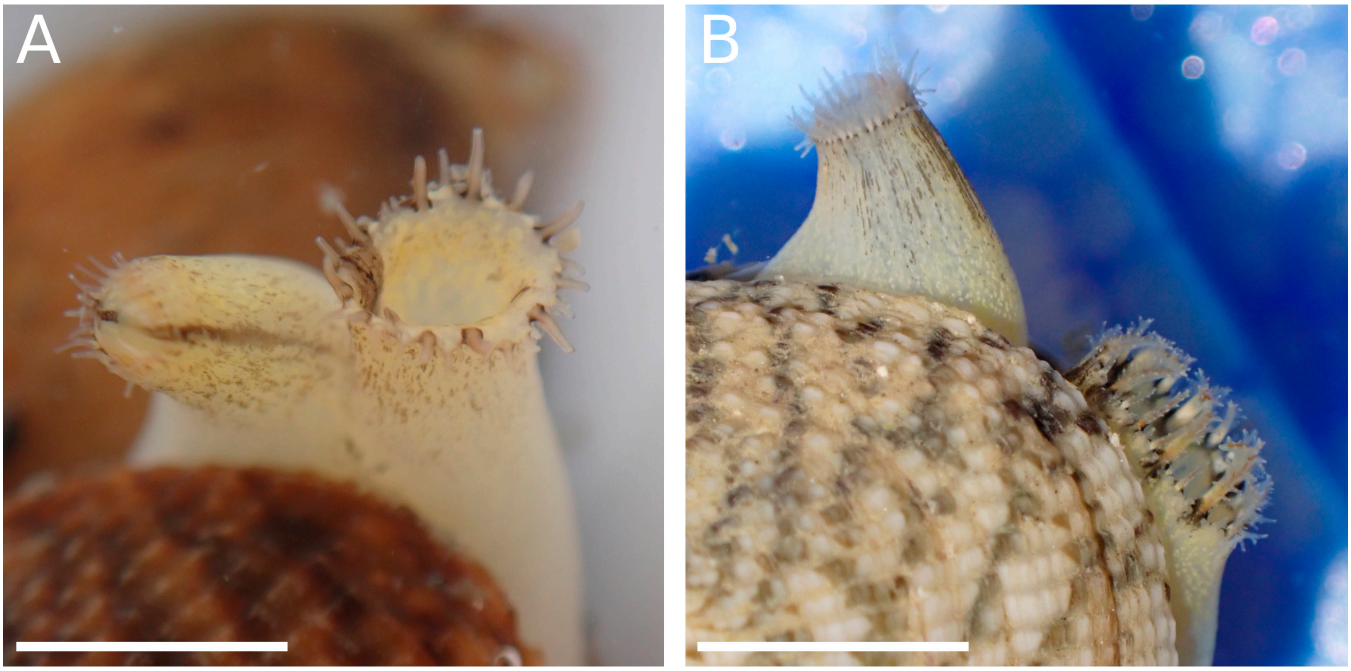

Figure S4. Representative siphonal morphology of *Ruditapes philippinarum* and *Ruditapes variegatus*. (A) *Ruditapes philippinarum* from the same commercial Chinese-origin lot used for the food-composition analysis. (B) *Ruditapes variegatus* from the same Ishigaki Island lot used for the food-composition analysis and COI sequencing. The two taxa were compared with reference to the diagnostic siphonal characters described by Sagara [15]. In *R. philippinarum*, the inhalant and exhalant siphons remain fused over most of their length and separate mainly toward the distal region, whereas in *R. variegatus* the two siphons separate closer to the base. Differences in the morphology of the marginal siphonal tentacles are also visible. Scale bars = 1 cm. Molecular identification of the Ishigaki *R. variegatus* material was independently supported by COI sequence analysis.
